# Di’ao Xinxuekang Capsule Improves the Anti-Atherosclerotic Effect of Atorvastatin by Downregulating the SREBP2/PCSK9 Signalling Pathway

**DOI:** 10.3389/fphar.2022.857092

**Published:** 2022-04-28

**Authors:** Jiyi Liang, Wei Li, Honglin Liu, Xiaofen Li, Chuqiao Yuan, Wenjun Zou, Liping Qu

**Affiliations:** ^1^ College of Pharmacy, Chengdu University of Traditional Chinese Medicine, Chengdu, China; ^2^ State Key Laboratory of Southwestern Chinese Medicine Resources, Chengdu University of Traditional Chinese Medicine, Chengdu, China

**Keywords:** DXXK, Atorvastatin (ATO), atherosclerosis, PCSK9 (proprotein convertase subtilisin kexin type 9), SREBP2, synergistic effect (combined treatment)

## Abstract

Statins are the first choice for lowering low-density lipoprotein cholesterol (LDL-C) and preventing atherosclerotic cardiovascular disease (ASCVD). However, statins can also upregulate proprotein convertase subtilisin/kexin type 9 (PCSK9), which in turn might limits the cholesterol-lowering effect of statins through the degradation of LDL receptors (LDLR). Di’ao Xinxuekang (DXXK) capsule, as a well-known traditional Chinese herbal medicine for the prevention and treatment of coronary heart disease, can alleviate lipid disorders and ameliorate atherosclerosis in atherosclerosis model mice and downregulate the expression of PCSK9. In this study, we further explored whether DXXK has a synergistic effect with atorvastatin (ATO) and its underlying molecular mechanism. The results showed that both ATO monotherapy (1.3 mg/kg) and ATO combined with DXXK therapy significantly lowered serum lipid levels and reduced the formation of atherosclerotic plaques and the liver lipid accumulation. Moreover, compared with ATO monotherapy, the addition of DXXK (160 mg/kg) to the combination therapy further lowered LDL-C by 15.55% and further reduced the atherosclerotic plaque area by 25.98%. In addition, the expression of SREBP2, PCSK9 and IDOL showed a significant increase in the model group, and the expression of LDLR was significantly reduced; however, there were no significant differences between the ATO (1.3 mg/kg) and the model groups. When ATO was combined with DXXK, the expression of LDLR was significantly increased and was higher than that of the model group and the expression of SREBP2 and PCSK9 in the liver was also significantly inhibited. Moreover, it can be seen that the expression of SREBP2 and PCSK9 in the combination treatment group was significantly lower than that in the ATO monotherapy group (1.3 mg/kg). Besides, the expression of IDOL mRNA in each treatment group was not significantly different from that of the model group. Our study suggests that DXXK might have a synergistic effect on the LDL-C lowering and antiatherosclerosis effects of ATO through the SREBP2/PCSK9 pathway. This indicates that a combination of DXXK and ATO may be a new treatment for atherosclerosis.

## Introduction

ASCVD is the leading cause of morbidity and mortality worldwide. Atherosclerosis (AS) is considered to be the basic pathogenesis of ASCVD and causes heart attacks, strokes and peripheral arterial disease (Libby et al., 2019; Cobos-Palacios et al., 2021). Dyslipidaemia is the main risk factor for ASCVD, especially an increase in LDL-C, which is closely related to the occurrence of ASCVD (Goldstein and Brown, 2015; Libby et al., 2019). Therefore, reducing LDL-C has become the primary goal for decreasing the risk of ASCVD in clinical practice. Statins, which are the hydroxy-3-methyl-glutaryl-CoA (HMG-CoA) reductase inhibitors, are able to inhibit the synthesis of endogenous cholesterol by competitively inhibiting HMG-CoA reductase, and accelerating the clearance of LDL-C in the circulation by upregulating the hepatic LDLR (Endo et al., 2004; Goldstein and Brown, 2015). Over the past 3 decades, evidence-based medicine studies have shown that statins can effectively lower serum lipid levels, reduce the occurrence of cardiovascular events, and slow the progression or even promote regression of coronary atherosclerosis (Michos et al., 2019; Wang et al., 2020). The latest 2021 European Society of Cardiology (ESC) Guidelines on cardiovascular disease prevention in clinical practice still recommend that statins as the first choice for lowering LDL-C and preventing ASCVD (Visseren et al., 2021).

However, statins are associated with an increased risk of diabetes mellitus and hepatic transaminase elevations, and might trigger myopathy (myalgia, fatigue, and rhabdomyolysis), which makes it impossible for some ASCVD patients unable to tolerate statins and forces them to reduce their doses or even stop the medication (Harrison et al., 2018; Li et al., 2020; Ruscica et al., 2022). Importantly, although statins might reduce LDL-C by 31%–63% on average at their highest approved doses (Tiwari and Khokhar, 2014), the reduction in LDL-C in response to statin therapy can vary by as much as 5%–70% across individuals. Even when compliance is taken into account, some specific individuals might hardly achieve the LDL-C target values. Only 16.2% of patients with heterozygous familial hypercholesterolemia (FH) achieved LDL-C management target of less than 100 mg/dl, even for strong statin or statins combined with ezetimibe (Reiner et al., 2013; Reiner and Tedeschi-Reiner, 2013; Ogura, 2018). The PCSK9 gene transcription can be activated by the sterol response element binding protein 2 (SREBP2), which could upregulate the LDLR gene as well. The main factors that limit the lipid-lowering efficacy of statins might be related to the upregulatory role of PCSK9 while upregulating the SREBP2 triggered by inhibiting HMG-CoA reductase and lipid-lowering effects. The increase in the level of PCSK9 in the circulation could mediate the increased degradation of LDLR, thereby reducing the uptake of LDL-C by hepatocytes even limiting the efficacy of statins, especially when PCSK9 was more significantly upregulated by statins in contrast to the LDLR induction and the net balance was in favor of PCSK9-induced degradation of LDLR over SREBP2 induction (Dong et al., 2010; Silbernagel et al., 2019; Macchi et al., 2021). Therefore, statins are often used in combination with other drugs to enhance the LDL-C lowering effect and reduce the dosage of statins, as well as decrease the risk of adverse effects in the clinic practice (Toth et al., 2016). The 2020 AACE/ACE Consensus Statement (Handelsman et al., 2020) recommends that statins can be used in combination with other lipid-lowering drugs (such as ezetimibe, and PCSK9 inhibitors) to improve their efficacy, but unfortunately, some lipid-lowering drugs (such as fenofibric acid) can also increase the expression of PCSK9, resulting in a limited degree of synergy when combined with statins (Khera et al., 2015). Although novel therapies such ANGPTL3 inhibition show a great clinical prospect, there is still a long way to go before marketing authorization (Olkkone et al., 2018). Among the different patient accessible combinations, that of PCSK9 inhibitors with statins is the most effective solution. Alirocumab, a fully human monoclonal antibody against PCSK9, in combination with high-intensity statins reduced LDL-C levels by 55% than with statin alone (66 vs. 103 mg/dl) (Sabatine et al., 2017; Schwartz et al., 2018). However, there is currently only a small number of small-molecule PCSK9 inhibitors, PCSK9 monoclonal antibodies are costly and their subcutaneous administration leads to poor compliance and inconvenience (Adorni et al., 2020). Therefore, it is important to develop low-cost PCSK9 inhibitors from natural medicines.

Di’ao Xinxuekang (DXXK) capsule is the rhizome extract of *Dioscorea nipponica* Makino and *Dioscorea panthaica* Prain et Burk and contains dioscin, diosgenin and other steroidal saponins. DXXK is used to prevent and treat coronary heart disease and other associated coronary artery diseases (CADs) (Qu et al., 2018; Li et al., 2021b). In Traditional Chinese Medicine theory, it has the effects of promoting blood circulation, and removing blood stasis which might explain its therapeutic effect in CADs. Modern pharmacological studies have shown that DXXK has the effects of anti-myocardial ischaemia, blood lipid lowering, anti-atherosclerosis, platelet aggregation prevention, inflammation inhibition, and antioxidative stress effects (Dong G. X. et al., 2017; Zhang weizhi and Chen, 2020; Li et al., 2021a). Our previous research has shown that DXXK can alleviate hyperlipidemia, fat accumulation, and atherosclerosis formation in ApoE^−/−^ mice fed a high-fat diet (HFD), and reduce the expression of liver PCSK9 and LDLR (Qu et al., 2018). Thus, in the present study, we explored whether DXXK can synergistically enhance the LDL-C-lowering effects and the antiatherosclerotic effect of atorvastatin (ATO) in ApoE^−/−^ mice fed a HFD and its possible molecular mechanism.

## Materials and Methods

### Materials and Reagents

Di’ao Xinxuekang capsule (DXXK) (batch number: 190801) used in our research was obtained by the Chengdu Diao Pharmaceutical Group Co., Ltd. (Chengdu, China). Atorvastatin calcium tablets (ATO) (batch number: EG 6200) was bought from Pfizer Pharmaceutical Co., Ltd. (Dalian, China). Colorimetric kits based on enzymatic reactions to determine the levels of mouse TC, TG, HDL-C, LDL-C, catalases (CAT), superoxide dismutase (SOD) and total antioxidant capacity (T-AOC) and the bicinchoninic acid (BCA) protein assay kit were purchased from Jiancheng Biotechnologies (Nanjing, China). The mouse PCSK9 ELISA kit (batch number: E-EL-M0634c) was purchased from Elabscience Biotechnology (Wuhan, China). The mouse TNF-α ELISA kit (batch number: 24116941013) was purchased from Boster Biological Technology Co., Ltd. (Wuhan, China). The Total RNA Kits and cDNA Synthesis Kits were bought from Foregene Biotechnology Co., Ltd. (Chengdu, China). Anti-LDL Receptor antibody (Cat#: ab52818) and anti-PCSK9 antibody (Cat#: ab185194) were purchased from Abcam (Cambridgeshire, United Kingdom). SREBF2 Rabbit pAb (Cat#: 513049) was obtained from Zen BioScience (Chengdu, China).

### Animals

Thirty-two 7-week-old male ApoE^−/−^ mice and eight wild-type C57BL/6J mice weighing 20–22 g were purchased from SiPeifu biotechnology Co., Ltd. (Beijing, China, No. SCXK- (jing) 2019-0010). All animals fed under specific pathogen free condition of temperature (20 ± 5°C), relative humidity (55 ± 5%), an alternating lighting (12 h light/12 h dark cycle), and free access to sufficient food and water. The experiment was approved by the Animal Ethics Committee of Chengdu University of Traditional Chinese Medicine, China. All procedures were performed according to the Guiding Principles for the Care and Use of Laboratory Animals of China.

### Experimental Design

All animals were fed a normal chow diet for 1 week prior to the experiment. Following this acclimation period, the C57BL/6J mice in the control group were fed a normal chow diet, and ApoE^−/−^ mice were randomly divided into four groups (*n* = 8): the model group, DXXK group (160 mg/kg), ATO group (1.3 mg/kg), and DXXK (160 mg/kg) + ATO (1.3 mg/kg) group. All ApoE^−/−^ mice were fed a HFD containing 40 kcal% fat and 0.21% cholesterol (D12079B, Open Source Diets, Research Diets, Inc.). Mice in the drug-treated groups were orally administered 160 mg/kg/d DXXK and/or 1.3 mg/kg/d ATO for 20 weeks, while those in the control and model groups were administered an equivalent volume of sterile water. The mice were fasted overnight and then anaesthetized with isoflurane, and the liver was collected from each mouse. The wet weight of the liver was recorded. The liver index was calculated with the following formula: liver index (%) = liver wet weight/mouse body weight × 100%.

### Assessment of Serum Lipids and Oxidative Stress

Blood samples were collected from the retro-orbital sinus after eyeball removal, followed by euthanasia, and then the serum was isolated by centrifugation of the blood samples at 3,500 × g (Sorvall ST-16R, Thermo) for 10 min at 4°C. The serum levels of TC, TG, LDL-C, and high density lipoprotein cholesterol (HDL-C) were measured with an enzyme colorimetric method according to the instructions provided with the kit manuals. The levels of total antioxidant capacity (T-AOC) and levels of superoxide dismutase (SOD), and catalase (CAT) in liver tissues were measured with detection kits based on the manufacturer’s instructions. The atherogenic index (AI) (Ramakrishna et al., 2017) was calculated with the following equation given below: AI = (TC-HDL-C)/HDL-C.

### Oil Red O Staining

As previously described (Shi et al., 2018), after blood samples were harvested, heart tissues containing the aortic arch were excised from the proximal aortic root to the iliac artery branch, and the liver tissues were dissected after removing external fatty deposits stripped. The heart and liver tissues were embedded with optimal cutting temperature (OCT) compounds and cut into 6-μm-thick and 10-μm-thick sections for histological analysis of aortic root atherosclerotic lesions and liver lipid accumulation. After oil red O staining, a digital trinocular camera microscope (BA210Digital, McAudi Industrial Group Co., Ltd.) was used to collect images of the slices, which were analysed by a blinded observer using the Image-ProPlus 6.0 software program (Media Cybernetics, United States).

### Haematoxylin-Eosin Staining

Liver tissues were fixed in 4% paraformaldehyde and then embedded in paraffin. Paraffin sections (5 μm thick) were cut and mounted on glass slides for haematoxylin-eosin (HE) staining as described previously (Yang F. et al., 2020). The histological images were captured with a light microscope (Olympus, Tokyo, Japan) at ×200 magnification.

### Enzyme-Linked Immunosorbent Assay

The levels of serum PCSK9 protein and TNF-α was measured by using enzyme-linked immunosorbent assay (ELISA) kit according to the manufacturer’s instructions. Color development at 450 nm was then measured using an ELISA autoanalyzer (Perkin Elmer, United States). In addition, a standard curve was generated for each assay plate by measuring the absorbance of serial dilutions of mouse PCSK9 and TNF-α at 450 nm.

### Quantitative Real-Time PCR

Total RNAs were extracted from the liver tissue by RNA simple Total RNA Kit. Total RNA (1 µg) were according to the Kit manufacturer’s protocol reverse-transcribed into cDNA using a cDNA Synthesis. Gene expression analysis was carried out using the Fast SYBR Green master mix and the CFX 96TM Real Time system (Bio-Rad, United States), as previously described (Harrison et al., 2018). The target genes were normalized with an endogenous reference gene β-actin. Relative gene expression was calculated using the comparative Ct method (2^−[△][△]Ct^). The primer sequences were shown in Table 1.

**TABLE 1:** The primer sequences for RT-PCR assay.

Gene	Forward primer (5′-3′)	Reverse primer (5′-3′)
LDLR	CCC​TTC​TCC​TTG​GCC​ATC​TA	TCG​ACT​TCT​CTA​GGC​TGT​GTG
PCSK9	TTT​GTC​TTC​GCC​CAG​AGC​AT	GTG​ACC​CTG​CCC​TCA​ATC​TC
SREBP2	GGGCTGTCGGGTGTCA	GGA​ACT​CTC​CCA​CTT​GAT​TGC​T
IDOL	CAG​GAG​CAG​ACA​AGG​CAT​ATC	GCT​CCT​TAT​GCT​TCG​CAA​CG
β-actin	CCA​CCA​TGT​ACC​CAG​GCA​TT	CAG​CTC​AGT​AAC​AGT​CCG​CC

### Western Blot

The liver tissue was lysed with RIPA lysis buffer (Solarbio, Beijing, China) containing 1mM PMSF, centrifuged at 12,000 rpm and 4°C for 10 min, the supernatant was aspirated, the centrifugation was repeated once, and the supernatant was quantified by the BCA Protein Assay Kit (Solarbio, Beijing, China). Equal-concentration samples were mixed with 5X loading buffer and boil it at 100°C for 10 min to denature the protein. The processed samples were separated on a 10% SDS-PAGE gel (80 V, 30 min, and then 100 V, 60 min), and the protein was transferred to the PVDF membrane according to the wet transfer protocol (270 mA, 90 min). The membranes were blocked in 5% skim milk solution at 37°C for 2 h. The membanes were washed one time with Tris-Buffered Saline-Tween 20 (TBST) buffer and incubated with a suitable primary antibodies (1:1,000) specific for LDLR, PCSK9 and SREBP2 at 4°C overnight, rinse with TBST four times, 10 min/time. Then incubate with secondary antibody (goat anti-rabbit) at 37°C for 1 h, rinse with TBST four times, 10 min/time. The immunoblotting is visualized by ultra-sensitive chemiluminescence. Quantitative analysis was performed using Image-ProPlus 6.0 software. The immunoblotting was visualized by the ultra-sensitive ECL chemiluminescence kit (Beyotime, Shanghai, China). Image-ProPlus 6.0 software was used to calculate the gray value of the protein bands, which represented the protein expression level.

### Statistical Analyses

All data were presented as mean ± standard deviation (SD). The SPSS software program (version 26.0; SPSS Inc., Chicago, IL, United States) was used for statistical analyses. Statistical differences between the groups were presented using one-way analysis of variance (ANOVA) or Kruskal-Wallis tests with the Bonferroni correction. *p* < 0.05 was considered statistically significant and *p* < 0.01 was highly significant. GraphPad Prism software for Windows (version 6.02; Inc., San Diego, CA, United States) were utilized for visible presentation of all results.

## Results

### Serum Lipid Levels of High-Fat Diet-Fed ApoE^−/−^ Mice

As shown in Table 2, compared with those of the control group, the serum TC, TG and LDL-C levels and the AI values of the model group were significantly increased (*p* < 0.01), and the serum HDL-C level was significantly decreased (*p* < 0.01). After treatment with DXXK alone (160 mg/kg) or ATO alone (1.3 mg/kg), the serum TG and LDL-C levels were significantly reduced (*p* < 0.05 or *p* < 0.01), and serum TC and HDL-C levels and AI value showed a reversal tendency. Treatment with DXXK+ATO showed similar effects and the serum TC and AI value showed a significant decrease compared with the model mice (*p* < 0.05 or *p* < 0.01). Moreover, the effect of lowering LDL-C was significantly greater than that of ATO monotherapy (*p* < 0.05), and the average level of serum LDL-C was further lowered from 14.50 ± 3.37 mmol/L in the ATO alone group to 11.63 ± 1.70 mmol/L in the combined DXXK+ATO group, which was a further reduction of 15.55%.

**TABLE 2:** Effect of DXXK, ATO, and their combination on serum lipid parameters and serum PCSK9.

Group	Serum TC (mmol/L)	Serum TG (mmol/L)	Serum HDL-C (mmol/L)	Serum LDL-C (mmol/L)	Atherosclerosis Index	Serum PCSK9 (ng/ml)
Control	3.94 ± 0.31∗∗	1.17 ± 0.14∗∗	3.39 ± 0.13∗∗	1.00 ± 0.28∗∗	0.32 ± 0.17∗∗	426.29 ± 34.83∗
Model	33.66 ± 5.04	3.61 ± 0.70	1.18 ± 0.35	18.44 ± 2.35	18.13 ± 2.69	502.72 ± 79.12
DXXK160	26.50 ± 1.36	2.09 ± 0.22∗∗	1.65 ± 0.39	13.17 ± 2.69∗∗	13.14 ± 2.23	353.29 ± 87.43∗∗
ATO1.3	26.40 ± 2.99	2.16 ± 0.61∗∗	1.53 ± 0.29	14.50 ± 3.37∗∗	13.22 ± 0.97	527.00 ± 82.38
DXXK160+ATO1.3	25.91 ± 2.91∗	2.12 ± 0.42∗∗	1.56 ± 0.35	11.63 ± 1.70∗∗^△^	11.16 ± 2.15∗∗	394.76 ± 63.29∗∗^△△^

Results are presented as the mean ± SD.

∗*p* < 0.05 vs. ApoE^−/−^ model group, ∗∗*p* < 0.01 vs. ApoE^−/−^ model group; ^∆^ *p* < 0.05 vs. ATO (1.3 mg/kg/day) group, ^∆∆^ *p* < 0.01 vs. ATO (1.3 mg/kg/day) group.

### Atherosclerotic Lesion Formation

**FIGURE 1:**
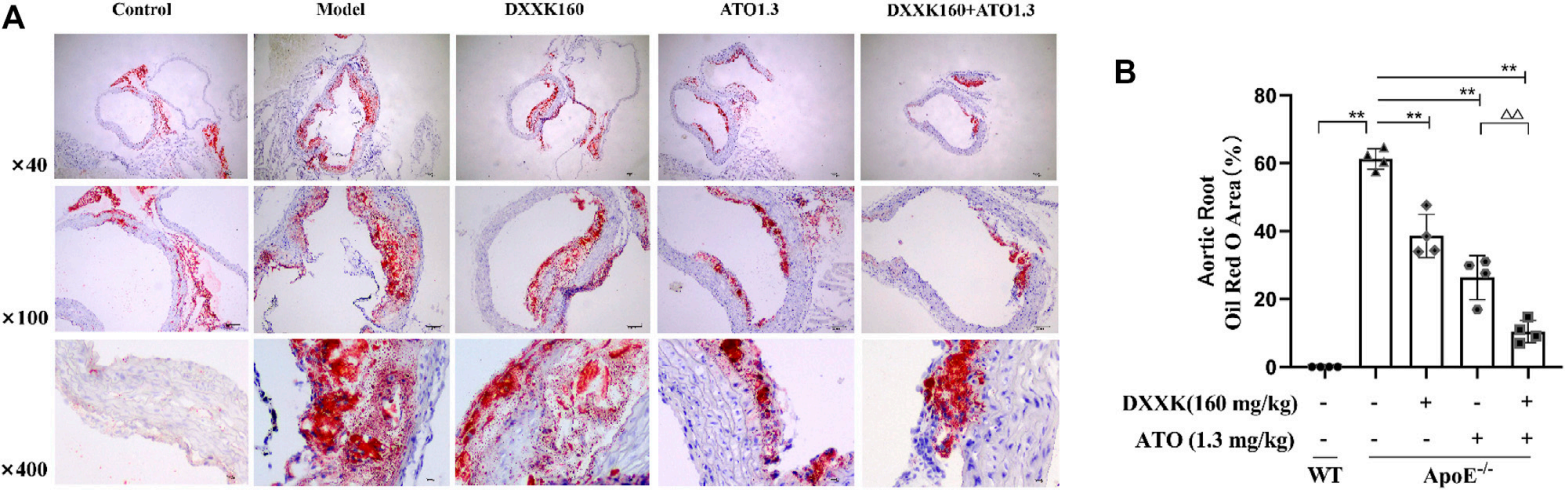
Effect of DXXK, ATO, and their combination on atherosclerotic lesion formation. **(A)** Representative images of mouse aortic root oil red O staining. **(B)** Quantification of the percentage of oil red O positive area in the cross section of the aortic root (*n*= 4). Results are presented as the mean ± SD. *∗∗p* < 0.01 vs. ApoE^−/−^ model group; ^*∆∆*^ *p* < 0.01 vs. ATO (1.3 mg/kg/day.) group.

After 20 weeks of treatment, oil red O staining was used to evaluate the development of atherosclerosis. Representative images of atherosclerotic lesions are shown in Figure 1A. A HFD induced the formation of atherosclerotic plaques in the aorta of ApoE^−/−^ mice, while treatment with DXXK alone, ATO alone or a combination of DXXK and ATO inhibited the formation of atherosclerotic lesions in the aortic root to varying degrees. To quantify the severity of the atherosclerotic lesions, we measured and calculated the percentage of oil red O-positive areas in the cross-section of the aortic root. As shown in Figure 1B, compared with those of the control group, the plaques in the aorta of the model group were significantly larger (*p* < 0.01), and the percentage of the lipid droplet expression area was 61.26 ± 3.02%. Compared with those in the model group, the plaque areas of the aortic root in the DXXK group, ATO group and DXXK+ATO group were significantly reduced (*p* < 0.01), and the percentages of lipid droplet expression area were 38.60 ± 6.35%, 26.34 ± 6.48% and 10.43 ± 3.28%, respectively. Moreover, treatment with the combination of DXXK and ATO further markedly reduced the AS plaque area of the aortic root by 25.98% compared with ATO monotherapy (*p* < 0.01).

### Liver Lipid Accumulation Induced by a High-Fat Diet

**FIGURE 2:**
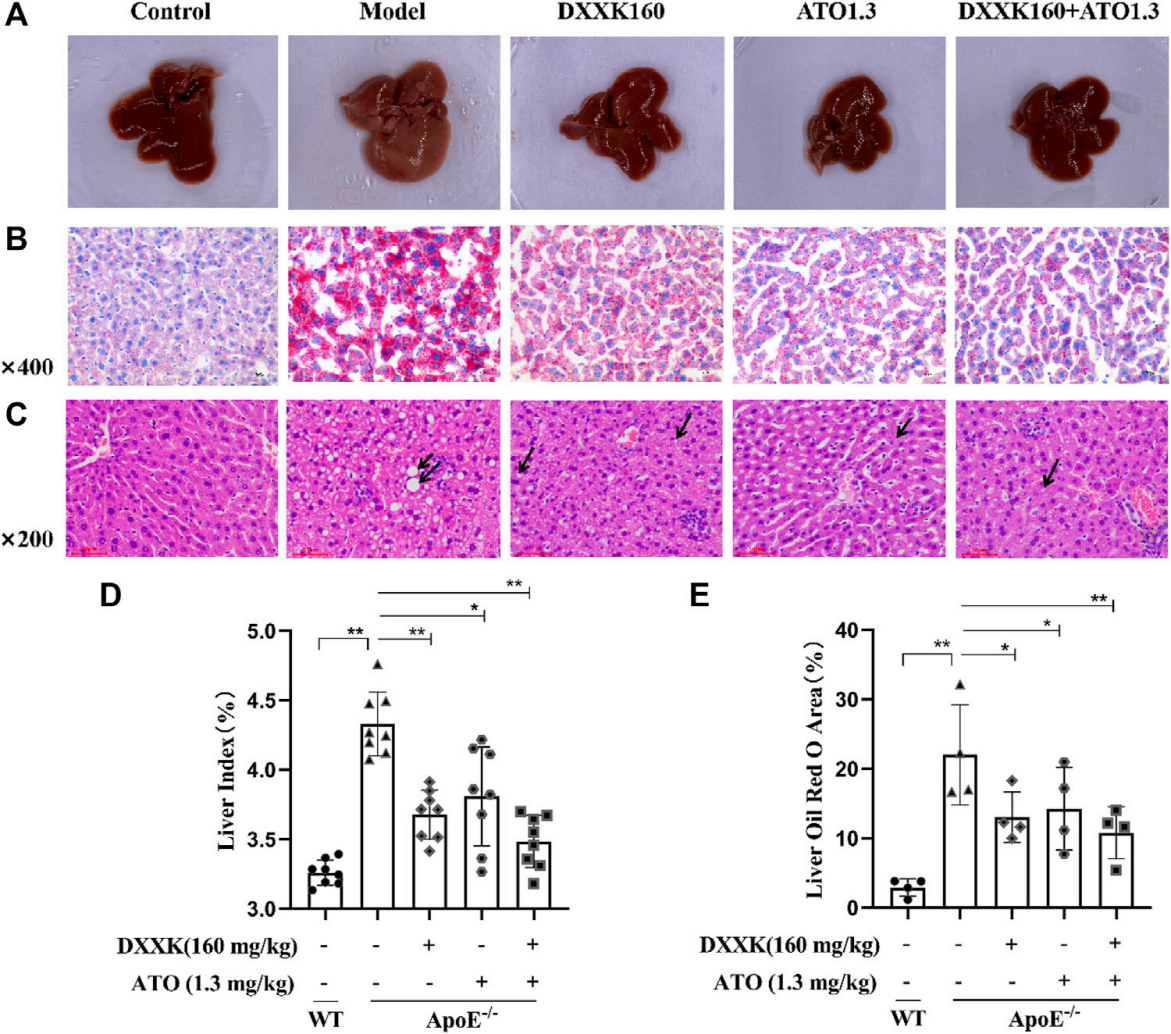
Effect of DXXK, ATO, and their combination on liver lipid accumulation and steatosis induced by HFD. **(A)** Representative liver photos. **(B)** Representative images of liver sections stained with Oil Red O. **(C)** Representative images of liver sections stained with H&E. **(D)** Liver index (n=8). **(E)** Quantification of the percentage of oil red O positive area of liver slices (n=4). Results are presented as the mean ± S.D. ^∗^ *p* < 0.05 vs. ApoE^−/−^ model group, ^∗∗^ *p* < 0.01 vs. ApoE^−/−^ model group.

The liver is the main organ responsible for lipid and lipoprotein metabolism, and atherosclerotic dyslipidemia is highly related to liver lipid accumulation (Xu et al., 2015). At the end of 20 weeks, HFD-fed ApoE^−/−^ mice exhibited a uniformly pale yellow liver indicating lipid accumulation in the liver (Figure 2A). The liver index values of each group was calculated and found that compared with the normal control group, the liver index values of mice in the model group was significantly increased (*p* < 0.01); compared with the model group, all drug-treated groups showed a reversal of the increase in liver index values caused by the HFD (*p* > 0.05, *p* < 0.01) (Figure 2D). Oil Red O staining (Figure 2B) and quantitative analysis of the lipid droplet area were performed to evaluate the liver lipid accumulation. As shown in Figure 2E, the accumulation of lipid droplets in the livers of HFD-fed ApoE^−/−^ mice was significantly higher than that of control mice (*p* < 0.01). The hepatocyte lipid droplet area ratios in the control group and model group were 2.89 ± 1.25% and 22.01 ± 7.22%, respectively. After treatment, lipid accumulation in the liver decreased significantly (*p* < 0.05 or *p* < 0.01). The hepatocyte lipid droplet cavity area ratios of the DXXK, ATO group and DXXK+ATO group were 13.04 ± 3.62%, 14.27 ± 5.95% and 10.79 ± 3.75%, respectively.

In addition, further HE staining results showed that the model group had obvious steatosis, with lipid droplets of different sizes, significant enlargement of liver cells, and partial stenosis or even atresia of the liver sinusoids. After treatment, the liver steatosis caused by the HFD improved to varying degrees. As shown in Figure 2C, mice in the DXXK group had moderately large and small vesicle-type steatosis, and there were many small round vacuoles in the cytoplasm; mice in the ATO group and the DXXK+ATO group showed mild vesicular steatosis, a small number of nearly round small vacuoles were seen in some mildly enlarged liver cells, and the hepatic sinus was clear.

### The Total Antioxidant Capacity and Levels of Superoxide Dismutase, Catalases in the Liver and Serum TNF-α

**FIGURE 3:**
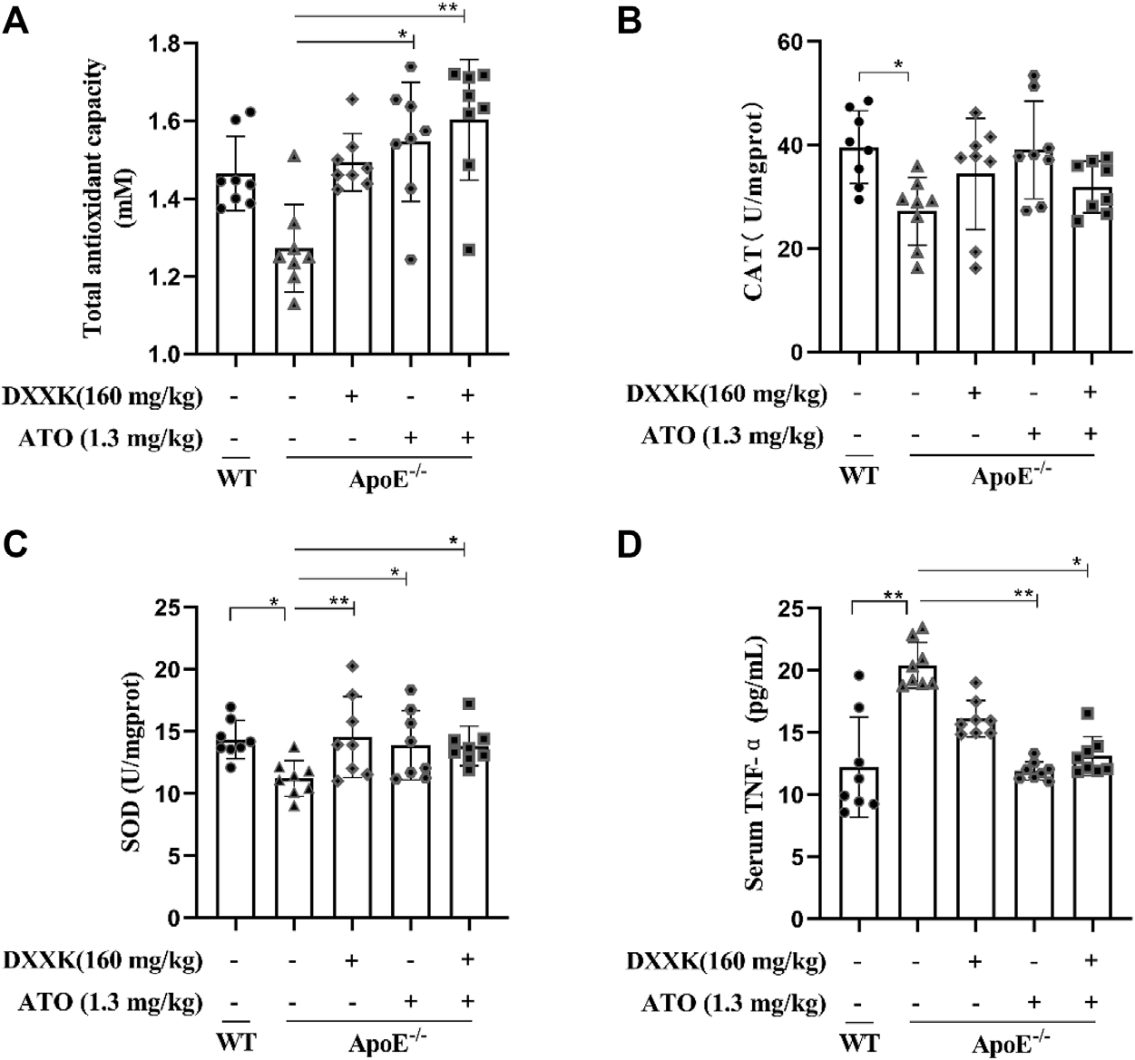
Effect of DXXK, ATO, and their combination on the antioxidant and anti-inflammatory abilities of ApoE^−/−^ mice induced by HFD. **(A)** T-AOC levels in the liver. **(B)** CAT levels in the liver. **(C)** SOD levels in the liver. **(D)** Serum level of TNF-α. (n=8) Results are presented as the mean ± SD. ^∗^ *p* < 0.05 vs. ApoE^−/−^ model group,*^∗∗^p* < 0.01 vs. ApoE^−/−^ model group.

Inflammation and oxidative stress are closely related to the occurrence and development of AS (Tabas et al., 2015; Cheng et al., 2017). We measured the T-AOC and levels of SOD and CAT in mouse livers and serum tumour necrosis factor α (TNF-α). As shown in Figures 3A–C, compared with those of the control group, the levels of CAT and SOD in the liver tissues of the model group were significantly decreased (*p* < 0.05), a declining trend was observed in T-AOC (*p* > 0.05). Compared with the model group, the level of SOD in each treatment groups exhibited significantly increased (*p* < 0.01 or *p* < 0.05), while the CAT level in each treatment group only had an increased trend, and the T-AOC of the ATO and DXXK+ATO groups were significantly increased (*p* < 0.05 or *p* < 0.01). The results of serum TNF-α measurement showed that feeding ApoE^−/−^ mice a HFD for 20 weeks significantly increased the serum TNF-α level (*p* < 0.01), and ATO monotherapy and DXXK+ATO therapy significantly inhibited the increase in TNF-α (*p* < 0.01 or *p* < 0.05) (Figure 3D).

### The Level of Serum Proprotein Convertase Subtilisin/Kexin Type 9 and mRNA and Protein Expression of Liver LDL Receptors and Proprotein Convertase Subtilisin/Kexin Type 9

**FIGURE 4:**
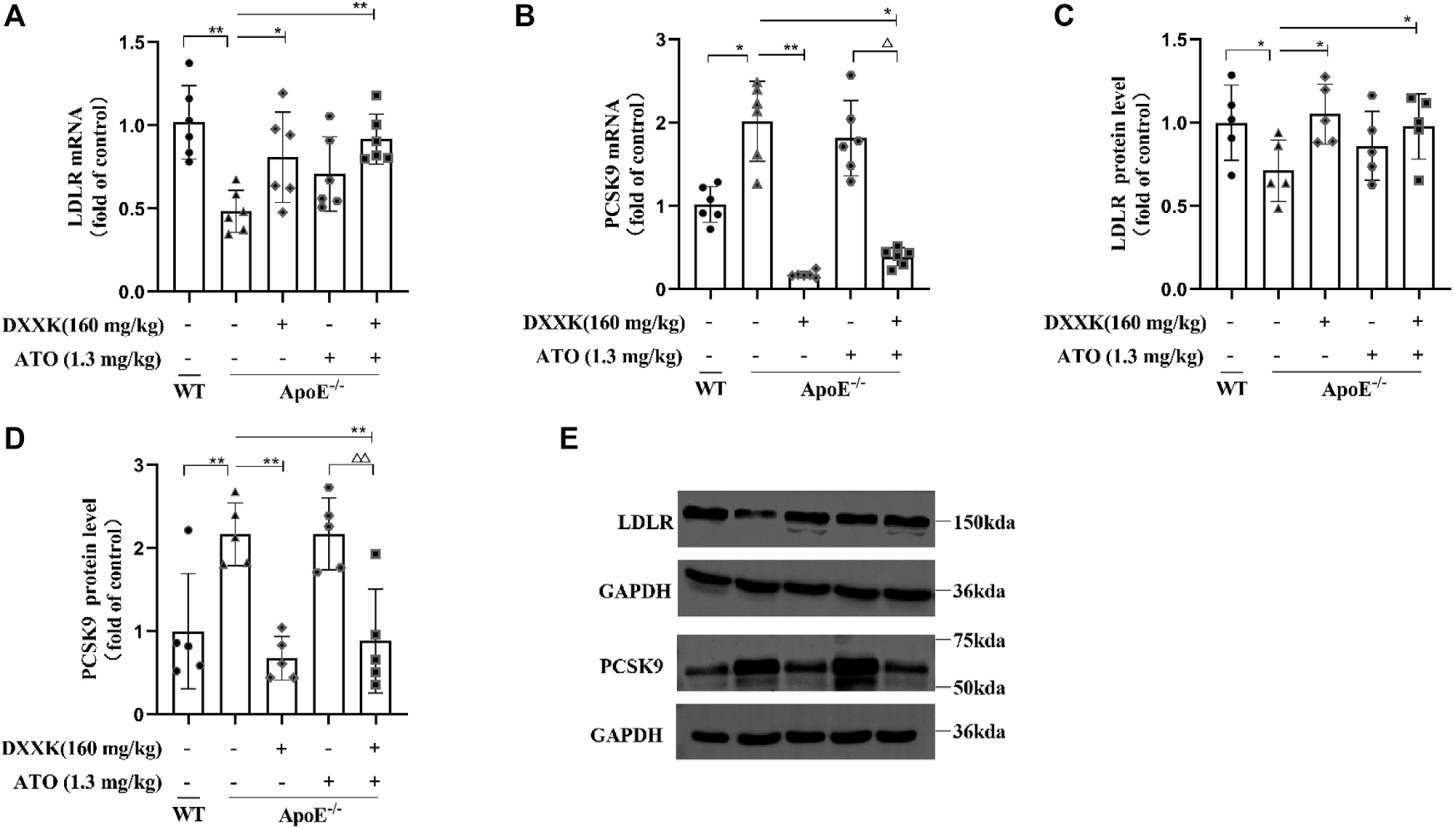
Effect of DXXK, ATO, and their combination on the levels of liver **(A)** LDLR mRNA and **(B)** PCSK9 mRNA, **(C)** LDLR and **(D)** PCSK9 protein expression in the liver (*n* = 5); **(E)** the representative blot bands. Results are presented as the mean ± SD. ^∗^ *p* < 0.05 vs. ApoE^−/−^ model group, ^∗∗^ *p* < 0.01 vs. ApoE^−/−^ model group, ^∆∆^ *p* < 0.01 vs. ATO (1.3 mg/kg/day.) group.

As shown in Figures 4A,B, compared with that of the control group, the liver LDLR mRNA level in ApoE^−/−^ mice fed a high-fat diet was significantly decreased (*p* < 0.01), and the PCSK9 mRNA level was significantly increased (*p* < 0.05). After treatment, the level of PCSK9 mRNA in the DXXK group and DXXK+ATO group was significantly decreased (*p* < 0.01 or *p* < 0.05), the level of LDLR mRNA was significantly increased (*p* < 0.05 or *p* < 0.01), and there was no significant change in the ATO group. Compared with that of the ATO group, the PCSK9 mRNA expression of the DXXK+ATO group was significantly lower (*p* < 0.05). Regarding to the protein expression, as shown in Figures 4C,D and Table 2, compared with that in the control group, the expression of liver LDLR protein in the model group was also significantly decreased (*p* < 0.05), and both liver PCSK9 protein expression and serum PCSK9 were significantly increased (*p* < 0.01). ATO monotherapy had no significant effect on the protein expression of PCSK9 and LDLR in the liver or serum PCSK9 levels in mice, while the expression of LDLR protein in the livers of the DXXK+ATO treatment group was significantly increased (*p* < 0.05) compared with that in the model group and both the expression of liver PCSK9 protein and serum PCSK9 were significantly lower (*p* < 0.01) than that in the model and ATO group.

### Expression of Liver SREBP2 mRNA and Protein and IDOL mRNA

**FIGURE 5:**
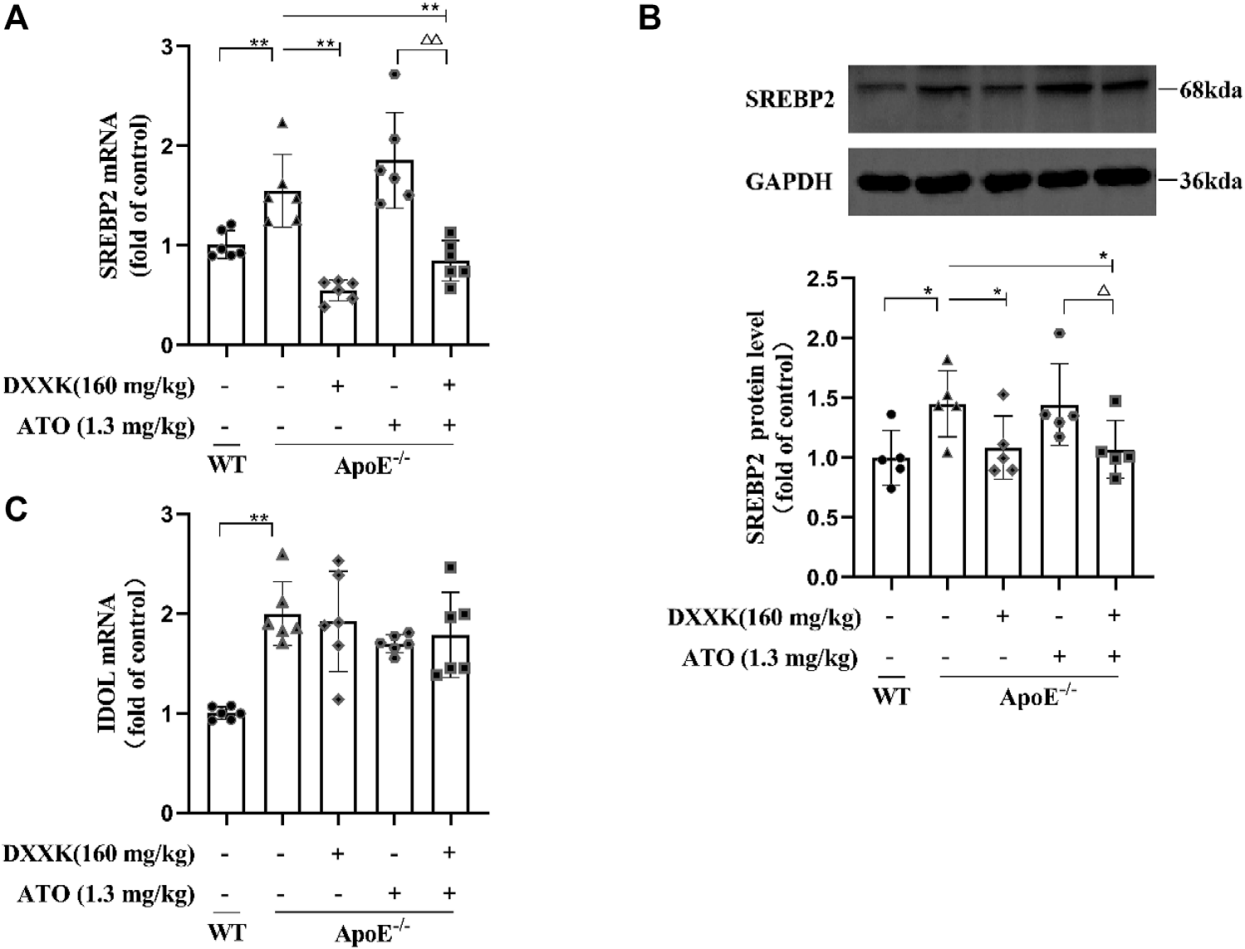
Effect of DXXK, ATO, and their combination on the levels of **(A)** SREBP2 mRNA, **(B)** SREBP2 protein expression in the liver (*n* = 5) and **(C)** IDOL mRNA. Results are presented as the mean ± S.D. ^∗^ *p* < 0.05 vs. ApoE^−/−^ model group, ^∗∗^ *p* < 0.01 vs. ApoE^−/−^ model group, ^∆^ *p* < 0.05 vs. ATO (1.3 mg/kg/day) group, ^∆∆^ *p* < 0.01 vs. ATO (1.3 mg/kg/day) group.

The expression of LDLR and PCSK9 is regulated by SREBP2, therefore, we quantified the expression of SREBP2 mRNA and protein in mouse liver. As shown in Figures 5A,B, compared with that of the control group mice, the mRNA and protein expression of SREBP2 in the livers of the model group mice was markedly increased (*p* < 0.01 or *p* < 0.05). In comparison with mice in the model group, mice in the ATO group showed no significant effect on the expression of SREBP2, while those in the DXXK+ATO group exhibited a further reduction in the mRNA and protein expression of SREBP2 compared with mice in the model group (*p* < 0.01 or *p* < 0.05). Moreover, the mRNA and protein expression of SREBP2 in mice treated with DXXK+ATO was also significantly lower than that in mice treated with ATO (1.3 mg/kg) monotherapy (*p* < 0.01 or *p* < 0.05). In addition, the expression of LDLR is also regulated by the posttranscriptional level of IDOL (Yang H. X. et al., 2020), thus we preliminarily measured the mRNA expression of IDOL in mouse liver. As shown in Figure 5C, compared with that of the control group, the IDOL mRNA level of the model group fed the HFD diet was significantly increased (*p* < 0.01), while the liver IDOL mRNA of each treatment group did not change significantly compared with the model group.

## Discussion

It is generally believed that the weakening of the clinical lipid-lowering efficacy of statins is related to the upregulation effect of PCSK9 expression by statins (Mayne et al., 2008; Gu et al., 2019). DXXK, a traditional Chinese medicinal product, has been used for the prevention and treatment of coronary heart disease in China for more than 30 years. Our previous study indicated that DXXK can inhibit the expression of PCSK9 and increase the expression of LDLR in the livers of APOE^−/−^ mice fed a HFD (Qu et al., 2018). Thus, in the present study, we further explored the effect of combination treatment with DXXK and ATO on AS model mice. Consistent with previously reported results (Qu et al., 2018), treatment with both DXXK and ATO alone significantly reduced serum TG and LDL-C levels, reduced the area of atherosclerotic lesions to varying degrees and reduced lipid accumulation in liver tissue. The combination therapy also showed a significant effect of lowering serum lipid, moreover, it had a more significantly greater effect on LDL-C reduction and inhibition of the formation of atherosclerotic plaques than ATO monotherapy, further reducing LDL-C by 15.55% and the AS plaque area by 25.98%. This suggests that DXXK has a synergistic effect with ATO in the treatment of AS.

The increased of PCSK9 expression and the subsequent increase in LDLR degradation are the main reasons for the limited efficacy of statins in further lowering LDL-C (Choi et al., 2017; Shah, 2019). In our study, ATO monotherapy (1.3 mg/kg) did not increase the expression of PCSK9 in model mice, and there was no significant difference in the protein expression of LDLR between the ATO and model groups. Several studies have shown that the effect of statin therapy on PCSK9 levels occurs in a clear dose–response manner (Nozue, 2017). Sultan Alvi et al. (2017) reported that atorvastatin at a dose of 10 mg/kg might increase the mRNA expression of PCSK9 in rats with hyperlipidaemia induced by consuming a high-fat diet for 30 days. Su et al. (2020) reported that atorvastatin at a dose of 7.2 mg/kg did not show significant changes in hepatic PCSK9 expression after 6 weeks of treatment in a hyperlipidaemic rat model. Guo et al. (2013) also reported that there was no significant increase in serum PCSK9 in patients with atherosclerosis treated with atorvastatin at 10 mg/day for 4 and 8 weeks, but atorvastatin at 20 mg/day significantly increased serum PCSK9 by 35% at 8 weeks. The dosage of ATO (1.3 mg/kg/day) in our study refers to the equivalent dosage of 10 mg/70 kg/day in adults. Therefore, we speculate that this lack of a noticeable change in PCSK9 after statin treatment in our study may be related to the dosage of the statin, the severity of the animal model and the course of the treatment. Although 1.3 mg/kg ATO did not increase PCSK9 in this study, it also did not significantly increase the level of LDLR either, which might limit the lipid-lowering and anti-AS effects to a certain extent. After treatment with a combination of ATO and DXXK, LDLR expression was significantly higher than that in the model group, and the expression of PCSK9 was significantly lower than that in the model group and was significantly lower than that in the model group and ATO group. This provides a biological basis for the synergistic effect of DXXK with statins.

SREBP2 is generally considered as a key regulator of LDLR and PCSK9. It can bind to the SRE-1 element of both the PCSK9 promoter and LDLR promoter and activate transcription; therefore the upregulation of SREBP2 will increase the transcription of PCSK9 and LDLR (Choi et al., 2017; Dong B. et al., 2017a). In our study, the expression of SREBP2 in the livers of APOE^−/−^ mice fed a HFD was significantly increased, along with an increase in the expression of PCSK9 and a decrease in that of LDLR. After treatment with ATO (1.3 mg/kg) alone, there was no significant effect on the expression of SREBP2, but treatment of ATO combined with DXXK further decreased the expression of SREBP2,which was significantly lower than that of the model group and the ATO group. This further explained why the expression of PCSK9 and LDLR did not change significantly when ATO was used alone but showed a reversal after treatment with DXXK+ATO. In addition, IDOL, an E3 ligase, can directly ubiquitinate LDLR and then degrade it in the lysosome (Lindholm et al., 2009). The expression of LDLR is also regulated by the posttranscriptional level of IDOL (Yang et al., 2018). Our results showed that the expression of IDOL mRNA in APOE^−/−^ mice fed a HFD was significantly increased. However, none of the treatments had a significant effect on the expression of IDOL mRNA.

In addition, chronic inflammation and increased oxidative stress are important atherogenic mechanisms, and oxidative stress and inflammation are considered to be key links in the pathogenesis of AS (Siti et al., 2015; Yang et al., 2018; Shah, 2019). Under oxidative stress, the content of antioxidant molecules such as SOD and CAT will decrease, directly damaging the vascular endothelium and inducing inflammation, thereby promoting the progression of atherosclerosis (Cheng et al., 2017; Forstermann et al., 2017). As a proinflammatory cytokine, TNF-α can activate inflammation-related signalling pathways in endothelial cells and macrophages and promote intracellular production and release of a large amount of proinflammatory substances (Zhu et al., 2018). In our study, we found that a long-term HFD led to an imbalance of oxidative stress in mice and the appearance of inflammatory response. Treatment with ATO and DXXK+ATO showed good anti-inflammatory and antioxidant effects, but there was no significant difference between the ATO monotherapy and combination therapy.

**FIGURE 6:**
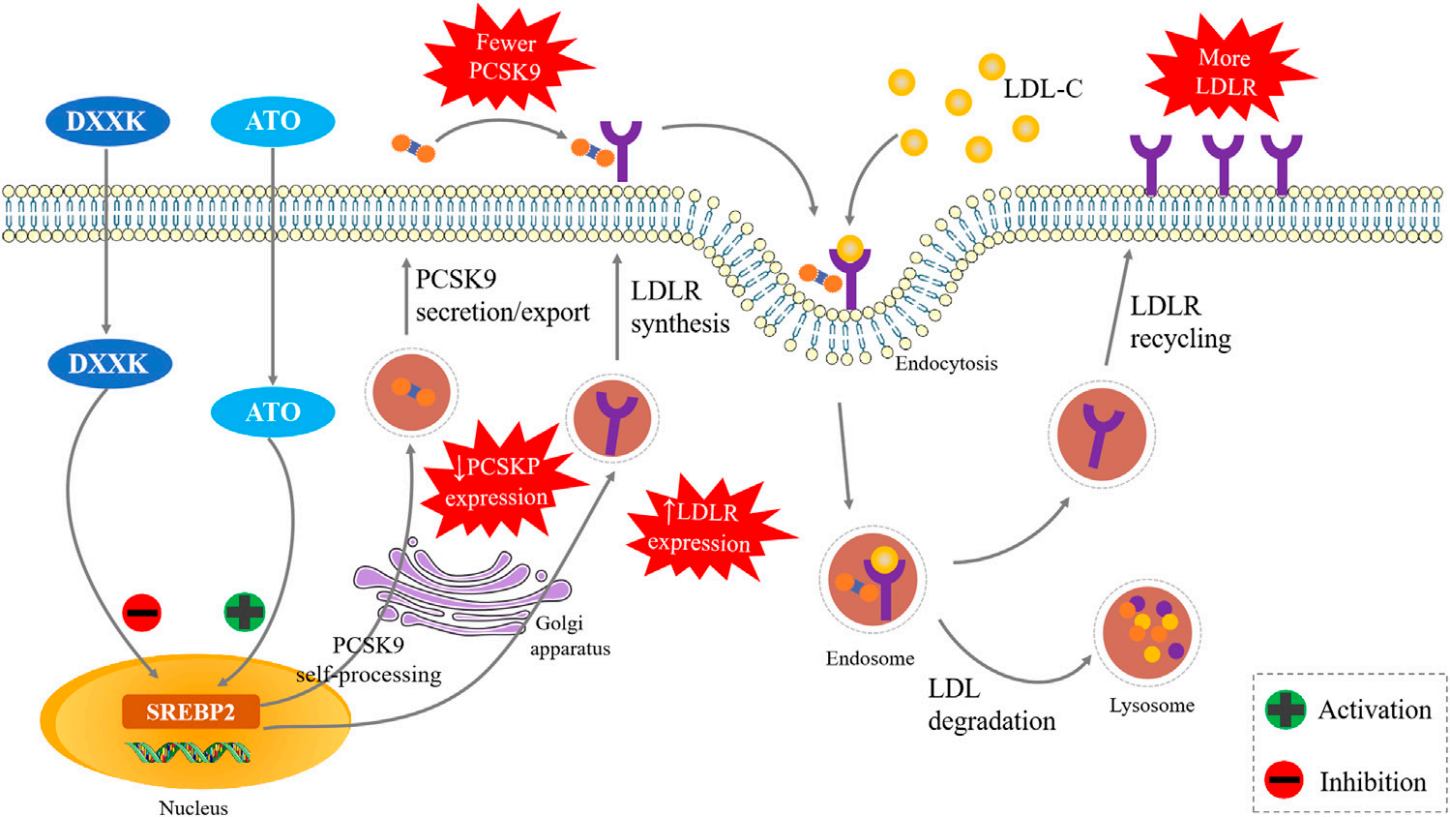
The DXXK Improves the Anti‐atherosclerotic Effect of ATO by SREBP2/PCSK9 pathway.

In summary, our experiments suggest that DXXK might synergistically enhance the effects of ATO in LDL-C lowering and its antiatherosclerotic, and this activity may be closely related to the SREBP2/PCSK9 signalling pathway (Figure 6). This suggests that the addition of DXXK might be helpful for enhancing the efficacy of statins in lowering LDL-C and exerting their anti-AS effect as well as reducing adverse reactions. As a marketed natural product, it is also more accessible to patients. In addition to SREBP2, HNF1α also plays an important role in activating the transcription of PCSK9 in hepatocytes (Dong B. et al., 2017a). Whether the HNF1α signal pathway contributes the synergistic effect of DXXK with statins is another promising point worth to be studied in the future.

## Data Availability

The original contributions presented in the study are included in the article/Supplementary Material, further inquiries can be directed to the corresponding authors.
